# Tissue quantitative RT–PCR test for diagnostic significance of cytomegalovirus infection in patients with inflammatory bowel disease and treatment response: Cytomegalovirus infection in patients with inflammatory bowel disease

**DOI:** 10.1097/MD.0000000000034463

**Published:** 2023-08-04

**Authors:** Burcu Ozdemir, Ali Atay, Meral Akdogan Kayhan, Yasemin Ozderin Ozin, Dilara Turan Gokce, Adalet Altunsoy, Rahmet Guner

**Affiliations:** a Department of Infectious Diseases and Clinical Microbiology Clinic, Ankara City Hospital, Ankara, Turkey; b Department of Gastroenterology, Ankara City Hospital, Ankara, Turkey; c Department of Infectious Diseases and Clinical Microbiology, University of Health Sciences Ankara City Hospital, Ankara, Turkey; d Department of Infectious Diseases and Clinical Microbiology Clinic, Ankara Yildirim Beyazit University, Ankara City Hospital, Ankara, Turkey.

**Keywords:** Crohn disease, cytomegalovirus colitis, inflammatory bowel diseases, real-time PCR, ulcerative colitis

## Abstract

Cytomegalovirus (CMV) is an opportunistic pathogen that exacerbates inflammatory bowel disease (IBD). There are no clear diagnostic criteria for CMV infection in IBD patients. The aim of this study was to evaluate the importance of the diagnosis of CMV infection with CMV-DNA polymerase chain reaction (PCR) in the colonic mucosa and the response to antiviral treatment. We retrospectively analyzed the clinical data of 30 patients with IBD (24 men, 6 women; median age: 42 years) who were hospitalized because of IBD exacerbation and whose samples were assessed by tissue CMV-DNA PCR positivity. Most of the IBD patients had ulcerative colitis (90%). The CMV-DNA PCR median value was 8848 copies/mL of tissue (range 90–242,936 copies/mL). Blood CMV-DNA PCR was found to be positive in a small group (33.3%, 10/30) of tissue CMV-DNA PCR-positive cases. immunohistochemistry tests were positive in only 5 of the 23 patients positive for CMV-DNA PCR in the colonic mucosa, and high remission (25/30, 83.3%) was detected with antiviral therapy. Recurrence of CMV colitis infection was observed in 9 of 25 patients who had remission with antiviral therapy. The tissue CMV-DNA PCR test was found to be more useful than blood CMV-DNA PCR and immunohistochemistry tests for diagnosing CMV colitis, and the tissue CMV-DNA PCR test enabled rapid and appropriate treatment.

## 1. Introduction

Cytomegalovirus (CMV), which belongs to the Herpesviridae family, is an opportunistic virus with both latent and reactive characteristics.^[1]^ Latent CMV infection may be reactivated as a result of immunosuppression. Patients with inflammatory bowel disease (IBD) are often immunosuppressed owing to malnutrition and immunosuppressive treatments.^[2]^

CMV that involves the colon may exacerbate infection and increase the incidence of toxic megacolon, colon perforation, and death.^[3]^ For this reason, early diagnosis and treatment of CMV colitis are important in cases of clinical exacerbation of patients with IBD. It is recommended that patients with colitis relapse should be screened for CMV and *Clostridium difficile* infection. The optimal method for detecting CMV colitis has unclear, but most experts agree it needs histology/immunohistochemistry rather than polymerase chain reaction (PCR) detection of CMV in the blood. The European Crohn and colitis organization guidelines (ECCO) recommend immediate antiviral treatment when CMV reactivation occurs in patients with acute severe colitis.^[4]^

The present study evaluated the importance of CMV infection diagnosis and antiviral treatment response using quantitative real-time PCR in the colonic mucosa of 30 IBD patients who were hospitalized for IBD exacerbations.

## 2. Materials and methods

### 2.1. Study design and participants

This retrospective study reviewed 30 IBD patients (aged > 18 years) who had clinical exacerbations and were hospitalized at Ankara City Hospital in Turkey between January 1, 2020, and September 31, 2022. Clinical flare-ups require admission and retreatment with steroids or new medications to control disease activity.

ECCO recommend that patients with colitis relapse were screened microbial testing for C. difficile and Cytomegalovirus infection.^[4]^ Hospitalized ulcerative colitis (UC) patients due to aggravated symptoms were obtained initial stool specimens culture including amebae, *C difficile* toxin assay to exclude infection.

Patients who had positive results on quantitative CMV-DNA RT–PCR of colon tissue biopsy samples were recorded and included in this study. The exclusion criteria were patients with negative CMV-DNA PCR of colon tissue. Patients were identified from the computerized databases of the Ankara City Hospital. The parameters included age, sex, comorbid diseases, potentially immunosuppressive medications, colon mucosa and serum quantitative CMV-DNA PCR, immunohistochemistry (IHC) test, treatment on admission and antiviral treatment.

#### 2.1.1. Cytomegalovirus real-time PCR.

CMV-DNA amplification from the extracted products was performed on the Rotor-Gene Q instrument (Qiagen) using the Artus CMV QS-RGQ Kit V1 kit. Blood and tissues samples were reported as copies/ml and copies/mg, respectively.

#### 2.1.2. Endoscopic and histopathological findings.

Findings from the colonoscopy or flexible sigmoidoscopy and pathology reports were reviewed. The gold standard for the diagnosis of CMV colitis was the histopathological findings. CMV-infected cells can be confirmed by immunohistochemistry staining.^[4]^ IHC test was performed in 23 of 30 patients included in our study.

Colitis was classified as proctitis (E1), left-sided (E2), and extensive (E3) in ulcerative colitis^[5]^ and ileal (L1), colonic (L2) and ileocolonic (L3) in Crohn disease^[6]^ according to the Montreal classifications. The severity of ulcerative colitis was classified according to the endoscopic Rachmilewitz index.^[7]^

#### 2.1.3. Antiviral therapy.

Patients were administered ganciclovir during hospitalization. The patients continued treatment with valganciclovir after hospital discharge. The total duration of antiviral therapy was 4 to 6 weeks. The clinical outcomes of the patients were reviewed after hospital discharge.

### 2.2. Ethical considerations

The study protocol and procedures for informed consent were approved by the Ankara City Hospital Ethical Committee (approval number: E2-22-2585, date: 12/10/2022).

### 2.3. Statistical Analysis

The data obtained were analyzed using the SPSS software for Windows, version 24 (IBM SPSS Statistics for Windows, version 24. Armonk, NY: IBM Corp.). Quantitative variables were presented as mean, standard deviation, and median values, while continuous data were presented as median, minimum, and maximum values because of skewed distributions; categorical data were described as frequencies and percentages. The relationships between categorical variables were evaluated using chi-square or Fisher exact test. Mann–Whitney or Kruskal–Wallis tests were used to compare continuous variables. Two-tailed *P* values of < .05 were defined as statistically significant.

## 3. Results

Thirty patients who were hospitalized for IBD exacerbations and were found to be CMV-DNA PCR-positive in the colonic mucosa were included in the study. The median age of the 30 patients was 42 years (range 19–76 years). Most of the patients (24/30.80%) were 30 years old or older and had ulcerative colitis (27/30, 90%). The extent of disease was pancolitis (11/30, 36.6%), left-sided colitis (8/30, 26.6%), proctitis (4/30, 13.3%), proctosigmoiditis (3/30, 10%), and extensive colitis (1/30, 3.3%). The mean endoscopic Rachmilewitz index was 10.1 ± 1.47. Comorbidities were present in 11 patients (36.7%) (Table 1).

**Table 1:** Clinical characteristics of 30 patients with inflammatory bowel disease.

Variables	
Age (median, min-max)	42 (19–76)
Sex (n, %)	
Male	24 (80)
Female	6 (20)
Extent of disease (n,%)	
Ulcerative colitis	
Pancolitis	11 (36.6)
Left-sided colitis	8 (26.6)
Proctitis	4 (13.3)
Proctosigmoiditis	3 (10)
Extensive colitis	1 (3.3)
Crohn Disease	
Ileocolonic	2 (6.6)
Colonic	1 (3.3)
Endoscopic index of Rachmilewitz (mean ± SD)	10.1 ± 1.47
Comorbidities	
Ankylosing spondylitis	1
AIDS	1
Hepatitis B virus infection	1
Hypertension	2
Primary biliary cirrhosis, liver transplantation	1
Coronary artery disease	2
Type 1 Diabetes mellitus	1
Breast cancer	1
Chronic obstructive pulmonary disease	1
Bening prostatic hyperplasia	2
Juvenile rheumatoid arthritis	1
Treatment on admission (n, %)	
Corticosteroid	
Corticosteroid alone	1 (3.3)
With azathioprine	9 (30)
With Adalimumab	2 (6.6)
Azathioprine	6 (20)
İnfliximab	3 (10)
Adalimumab	3 (10)
(5-ASA)	3 (10)
No therapies	3 (10)
Ratio undergoing colectomy	2

AIDS = acquired immunodeficiency syndrome.

Most patients (24/30, 80%) had been treated with immunosuppressive therapy. Of the 30 patients, 3 (10%) were treated with 5-aminosalicylic acid (5-ASA), 3 (10%) with adalimumab, 3 (10%) with infliximab, 6 (20%) with azathioprine, 9 (30%) with azathioprine with corticosteroids, 2 (6.7%) with adalimumab with corticosteroids and 1 (3.3%) with corticosteroids when visiting our institution. Three patients (10%) were not treated. Two patients (6.6%) underwent colectomy during the observation period. The clinical characteristics of the 30 patients are summarized in Table 1.

**Figure 1.**
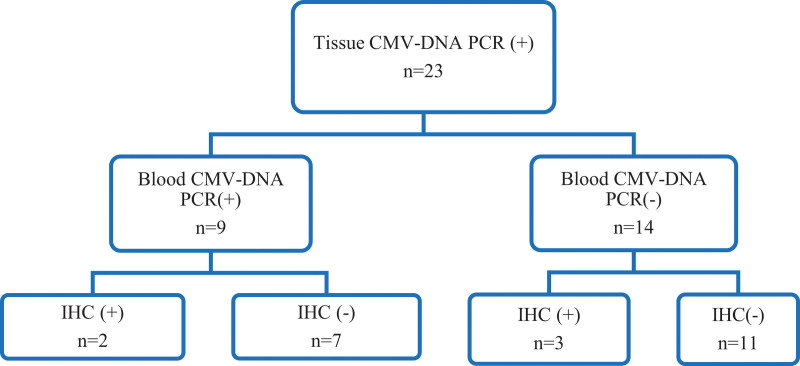
Distribution of the results of tissue CMV-DNA PCR, blood CMV-DNA PCR, and IHC test. CMV = Cytomegalovirus, IHC = immunohistochemistry, PCR = polymerase chain reaction.

Thirty patients who were CMV-DNA PCR-positive in the colonic mucosa were included in the study. The median value of CMV-DNA PCR was 8848 copies/ml (range 90-242,936 copies/ml) in the tissue. CMV-DNA PCR was positive in a small proportion (33.3%, 10/30) of tissue CMV-DNA PCR-positive cases. The median CMV-DNA PCR value in the blood was 0 copies/ml (min-max: 0–35,492 copies/ml). The IHC test results were positive in only 5 (21.7%) of the 23 patients who were positive for CMV-DNA PCR in the colonic mucosa (Fig. 1). Patients with positive and negative blood viral load results were divided into 2 groups. The demographic characteristics were compared in terms of tissue viral load, IHC, recurrence, and recovery, and no statistically significant differences were found (Table 2).

**Table 2:** Comparison of clinical and laboratory parameters between inflammatory bowel disease patients who had CMV-DNA PCR-positive in inflamed mucosa with and without detectable blood CMV-DNA PCR.

	BVL negative	BVL positive	*P* value
	n = 20	n = 10	
Age (median, min-max)	41 (21-76)	49 (19-72)	.775
Age ≥ 30 (n, %)	16 (80)	8 (80)	1.000
Gender (male)	15 (75)	9 (90)	.633
IHC			
Positive	3 (21,4)	2 (22.2)	1.000
Negative	11 (78,6)	7 (77.8)	
TVL (median, min-max)	13,180 (96–234,375)	3177 (90–242,936)	.159
≥10.000	10 (50)	3 (30)	.440
<10.000	10 (50)	7 (70)	
≥250	17 (85)	7 (70)	.372
<250	3 (15)	3 (30)	
Remission			
(+)	16 (80)	9 (90)	.640
(−)	4 (20)	1 (10)	
Relapse			
(+)	7 (35)	3 (30)	1.000
(−)	13 (65)	7 (70)	
Endoscopic index of Rachmilewitz	10 (8–12)	10 (7–12)	.419

BVL = blood viral load, CMV = Cytomegalovirus, IHC = immunohistochemistry, PCR = polymerase chain reaction, TVL = tissue viral load.

**Figure 2.**
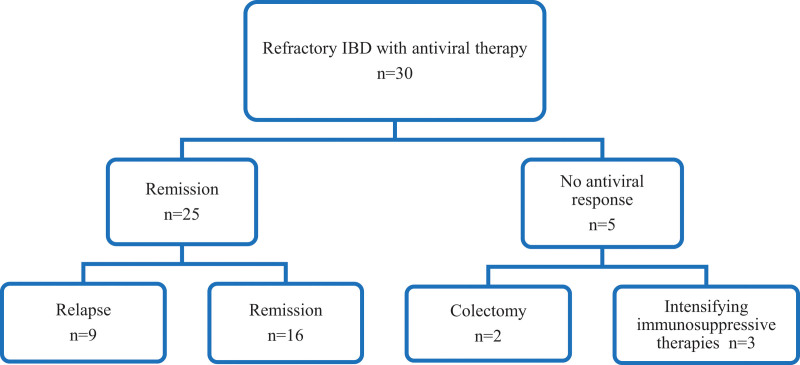
Clinical course of 30 patients who had CMV-DNA in inflamed mucosa was positive with inflammatory bowel disease (IBD) refractory columns show final outcomes. CMV = Cytomegalovirus.

All patients with positive CMV-DNA PCR results in the colonic mucosa were treated with antiviral therapy. Of the 30 patients who received antiviral therapy, 25 (83.3%) went into remission, and of the remaining 5 patients, 2 of them (16.7%) received colectomy. Three of them went into remission after treatment with more intense immunosuppressive therapies after the CMV-DNA PCR became negative with antiviral therapy. In the follow-ups, recurrence of CMV colitis infection was observed in 9 of 25 patients who were found to be in remission with antiviral treatment (Fig. 2). Those with relapse and continued remission during follow-up were divided into 2 groups, which are compared in Table 3. No statistically significant differences were found between the groups in terms of the endoscopic Rachmilewitz index, age, tissue viral load, blood viral load, and IHC (Table 3).

**Table 3:** Comparison of clinical and laboratory parameters according to remission and relapse at IBD cases.

	Remission	Relapse	P value
	(n = 16)	(n = 9)	
Age (median, min-max)	40 (19–71)	46 (33–72)	.202
Age ≥ 30 (n, %)	12 (75)	9 (100)	.260
TVL (median, min-max)	8018 (96–242,936)	16,404 (156–86,667)	.734
TVL (n, %)			.434
≥10,000	6 (37.5)	5 (55.6)	
<10,000	10 (62.5)	4 (44.4)	
BVL (median, min-max)	0 (0–14,607)	0 (0–35,492)	.895
IHC (n, %)			
Positive	2 (18.2)	1 (12.5)	1.000
Negative	9 (81.8)	7 (87.5)	
Endoscopic index of Rachmilewitz	10 (7–12)	10 (8–12)	.773

BVL = blood viral load, IBD = inflammatory bowel disease, IHC = immunohistochemistry, TVL = tissue viral load.

## 4. Discussion

CMV disease frequently occurs in immunocompromised patients, including transplant recipients, patients with acquired immunodeficiency syndrome, and patients receiving chemotherapy or steroids.^[8]^ CMV colitis is most commonly associated with UC, followed by Crohn disease (CD), immunosuppression, and hematological malignancy.^[9–11]^ In the present study, some of the patients had comorbidities that caused additional immunosuppression in addition to IBD, such as acquired immunodeficiency syndrome, a history of liver transplantation, and breast cancer. Previous studies have reported that the prevalences of CMV colitis and CMV disease are significantly higher in UC than in CD. Kim et al^[12]^ reported that all IBD cases with CMV colitis were UC (n = 20). No CMV colitis was detected in CD. Gauss et al^[13]^ reported that the rate of CMV colitis was higher in UC than in CD (80.9% vs 19.1%, respectively). Most of the patients had UC (27/30, 90%), and only 10% (3/30) were diagnosed with CD in the present study.

In a meta-analysis that included a total of 2099 patients with UC and investigated CMV risk factors, it was shown that the presence of pancolitis may increase CMV reactivation and that the risk of CMV reactivation is 2.108-fold higher in patients with pancolitis than in those with limited left colon lesions.^[14]^ In the present study, most patients had pancolitis (11/30, 36.6%) and left-sided colitis (8/30, 26.6%). In another similar study, most of the CMV-DNA PCR-positive tissue patients (76.4%) had pancolitis and left-sided colitis (23.5%).^[15]^ Studies have reported that the risk factors for IBD patients with CMV colitis include being 30 years or older and being refractory to immunosuppressive treatments such as corticosteroids or anti-TNF immunomodulatory therapy.^[13,16]^ In the present study, the median age of the patients was 42 years, and most of them (24/30, 80%) were aged 30 years or older. This finding is consistent with previous studies reporting that patients with IBD and CMV infection are older.^[17,18]^ Additionally, most of the patients (24/30, 80%) who were diagnosed with CMV colitis in our study had been treated with corticosteroid and immunosuppressive treatment and were refractory to these treatments. Most of the immunosuppressive treatments (18/24,75%) that were administered were azathioprine and corticosteroids. In a meta-analysis, azathioprine treatment was found to be a risk factor for CMV reactivation in patients with UC, and corticosteroid treatment increased the risk of CMV reactivation by 4.175-fold.^[14]^ A meta-analysis of 1306 patients with corticosteroid-refractory ulcerative colitis reported that CMV infection is an independent risk factor for corticosteroid resistance.^[19]^ Severe ulcerative colitis refractory to corticosteroid was confirmed for CMV infection in the ECCO guidelines, and more aggressive treatment is recommended.^[20]^

Clinical and endoscopic findings are insufficient for the diagnosis of CMV colitis in patients with IBD who are refractory to immunosuppressive therapy because of nonspecific findings. Quantitative CMV-DNA PCR in tissue and blood is the most commonly used technique for the diagnosis of CMV infection.^[9]^ CMV-DNA PCR testing in the blood can diagnose CMV viremia and potentially detect CMV throughout the body; however, it does not indicate definitive CMV infection in the colonic mucosa. Therefore, CMV-DNA positivity should be investigated using PCR in colonic mucosa samples for diagnosis. In the present study, most of the patients (66.6%, 20/30) who were found to have positive CMV-DNA PCR in the colonic mucosa had negative CMV-DNA PCR in the blood. Negative CMV-DNA PCR results in blood tests do not exclude the diagnosis of CMV colitis in patients with IBD.^[21]^ In a similar study, the blood CMV-DNA PCR results were negative in 33.3% of cases with positive tissue CMV-DNA PCR results.^[22]^ We suggest that all IBD patients who are hospitalized for exacerbations be evaluated using tissue CMV-DNA PCR in colonic biopsy specimens for the diagnosis of CMV colitis. CMV-DNA PCR positivity can be detected in tissue, although CMV-DNA PCR is negative in the blood.

The ECCO guidelines recommend tissue CMV-DNA PCR and IHC tests to exclude the diagnosis of CMV infection in patients with colitis.^[23]^ IHC testing of colonic biopsy specimens could be accepted as the gold standard for CMV colitis.^[24,25]^ However, some previous studies have reported that the sensitivity of the IHC test was not as good as that of the CMV-DNA PCR test in tissue.^[15,26]^ In the present study, the IHC test was positive in only 5 (21.7%) of the 23 patients positive for CMV-DNA PCR in the colonic mucosa. Alacam et al^[22]^ reported that 35.1% of cases with CMV-DNA PCR-positive tissue were found to be IHC positive. Although IHC is the gold standard, it alone may not be sufficient for diagnosis. IHC tests should be evaluated together with molecular tests for the diagnosis of CMV disease, as suggested by the ECCO guidelines.

The exclusion of CMV colitis from IBD exacerbations is important for the treatment approach. In IBD patients with CMV colitis, antiviral therapy should be promptly administered, and immunosuppressive drugs should be withdrawn. In contrast, in IBD patients without CMV colitis, more intensive immunosuppressive therapy is needed. CMV colitis can cause complications or mortality when antiviral therapy is not used. In previous studies, severe UC refractory to immunosuppressive treatments with CMV colitis achieved remission with antiviral therapy.^[27,28]^ However, when CMV infection is confirmed in patients with IBD, there is no evidence of the effectiveness of antiviral therapy for CMV infection. Meta-analyze reported conflicting results about the efficacy of antiviral therapy in patients with IBD.^[29]^ Matsuoka reported that the presence of CMV in severe UC is transient and may disappear without antiviral therapy.^[30]^ Studies reported that antiviral therapy response rate with in patients who have reactivation of CMV is %72 (range 50%–83%).^[15,31–33]^ These conflicting results are considered to have occurred because of the different clinical conditions and treatment indications in each study. In the present study, all of the patients with positive CMV-DNA PCR in the colonic mucosa were treated with antiviral therapy, and a high remission rate was achieved (25/30, 83.3%). Of the remaining 5 patients, 2 (16.7%) received a colectomy. Relapse was observed in 9 of 25 cases with remission during follow-up. In a similar study, it was reported that a high remission rate was obtained in 83.3% (10/12) of the cases with positive CMV-DNA PCR in the inflamed colonic mucosa in UC patients resistant to immunosuppressive treatments, and colectomy was performed in 2 cases.^[15]^

## 5. Conclusion

Severe IBD, pancolitis, immunosuppressive agents, glucocorticoids, azathioprine, and age > 30 years are risk factors for CMV reactivation in patients with IBD. Patients with these risk factors should be evaluated for CMV reactivation to ensure early detection and timely antiviral treatment. For patients with IBD who are hospitalized for exacerbations, endoscopic examination, biopsy, PCR and IHC in the tissue are important. The CMV-DNA PCR test in the colonic mucosa is more useful than blood CMV-DNA PCR and IHC tests in the diagnosis of CMV infection. This approach has enabled rapid and appropriate treatment. The present study also had limited characteristics because of the small amount of data. But we only included CMV-PCR-positive cases in tissue for evaluate the treatment response. Because diagnostic criteria for CMV infection in IBD patient is unclear. ECCO recommends CMV-DNA PCR in tissue and the histopathological findings for diagnosis of CMV colitis. Another limitation is that posttreatment findings (colonoscopy, histopathological findings) were not available due to the retrospective nature of our study. To determine the contribution of this method in improving the prognosis of IBD patients with CMV infection, further studies are needed to provide clear diagnostic criteria and standard treatment methods.

## Author contributions

**Conceptualization:** Burcu Ozdemir, Meral Akdogan Kayhan, Yasemin Ozderin Ozin, Dilara Turan Gokce, Adalet Altunsoy, Rahmet Guner.

**Data curation:** Burcu Ozdemir, Ali Atay, Meral Akdogan Kayhan, Yasemin Ozderin Ozin, Dilara Turan Gokce, Adalet Altunsoy, Rahmet Guner.

**Formal analysis:** Burcu Ozdemir, Meral Akdogan Kayhan, Yasemin Ozderin Ozin, Adalet Altunsoy, Rahmet Guner.

**Funding acquisition:** Burcu Ozdemir.

**Investigation:** Burcu Ozdemir.

**Methodology:** Burcu Ozdemir.

**Project administration:** Burcu Ozdemir, Ali Atay.

**Resources:** Burcu Ozdemir.

**Software:** Burcu Ozdemir.

**Supervision:** Burcu Ozdemir.

**Validation:** Burcu Ozdemir.

**Visualization:** Burcu Ozdemir.

**Writing – original draft:** Burcu Ozdemir, Ali Atay.

**Writing – review & editing:** Burcu Ozdemir.
